# Acute coronary syndrome patterns in the Young: risk factor profile and in-hospital outcomes in a tertiary referral hospital in Kenya

**DOI:** 10.1186/s12872-024-03832-z

**Published:** 2024-04-03

**Authors:** Nadeem Kassam, Mzee Ngunga, Mohamed Varwani, Miriam Msunza, Mohamed Jeilan

**Affiliations:** Department of cardiology, Aga Khan University Kenya, P.O. Box 30270 – 00100, Nairobi, Kenya

**Keywords:** Acute Conary syndrome, Young, Kenyans, Hospital outcomes, Risk factor profile, Mortality

## Abstract

**Introduction:**

Acute coronary syndrome (ACS) accounts for coronary artery disease (CAD) –related morbidity and mortality. There has been growing concern about the rising incidence of ACS among young individuals globally both in developed and developing countries, including Sub-Saharan Africa. This group’s phenotypic characteristics; risk factors and clinical outcomes are not well described. contextual and regional studies are necessary to understand the magnitude of ACS among young Individuals and help highlight challenges and opportunities for improved ACS outcomes in the region. The study aimed to describe the demographic and clinical characteristics of young individuals hospitalized with ACS and report on in-hospital outcomes.

**Methodology:**

This single-center retrospective study was conducted at the Aga Khan University Hospital, Nairobi. Medical records of all young individuals hospitalized with ACS from 30th June 2020 to 1st May 2023 were reviewed. We defined young individuals as 50 years or below. Categorical variables were reported as frequencies and proportions, and compared with Pearson chi- square or Fisher’s exact tests. Continuous variables were reported as means or medians and compared with independent t-tests or Mann-Whitney U tests. P- value < 0.05 was considered statistically significant.

**Results:**

Among 506 patients hospitalized with ACS, (*n* = 138,27.2%) were aged 50 years and below. The study population was male (*n* = 107, 79.9%) and African(*n* = 82,61.2%) predominant with a median age of 46.5 years (IQR 41.0–50.0). Hypertension (*n* = 101,75.4%) was noted in most study participants. More than half of the cohort were smokers (*n* = 69,51.5%) having a family history of premature ASCVD(*n* = 70,52.2%) and were on lipid-lowering therapy(*n* = 68,50.7%) prior to presentation. ST-segment–elevation myocardial infarction (STEMI) was the most common clinical manifestation of ACS (*n* = 77, 57.5%). Of the significant coronary artery disease (*n* = 75,56.0%), the majority of the individuals had single vessel disease (*n* = 60, 80%) with a predilection of left anterior deciding artery(*n* = 47,62.6%). The Main cause of ACS was atherosclerosis (*n* = 41,54.6%). The mean left ventricular ejection fraction was 46.0 (± 12.4). The in-hospital mortality was (*n* = 2, 1.5%).

**Conclusion:**

This study highlights that young individuals contribute to a relatively large proportion of patients presenting with ACS at our center. The most common presentation was STEMI. The principal cause was atherosclerosis. The findings of this study highlight the importance of developing systems of care that enable the early detection of CAD. Traditional cardiovascular risk factors were prevalent and modifiable, thus targets of intervention.

**Supplementary Information:**

The online version contains supplementary material available at 10.1186/s12872-024-03832-z.

## Introduction

Cardiovascular diseases (CVDs) are the leading cause of mortality and loss of Disability Adjusted Life Years (DALYs) worldwide [1]. A substantial burden of it occurs in Low and Middle- income countries (LMICs) [2]. In Africa, CVDs are the most significant contributor to the total Non-Communicable Disease (NCD) burden [3, 4]. Acute coronary syndrome (ACS) accounts for coronary artery disease (CAD) related morbidity and mortality [3]. Growing evidence suggests that the incidence of ACS is increasing in Sub-Saharan Africa due to poorly controlled cardiovascular risk factors and rapid urbanization [5, 6]. While the incidence of ACS among the elderly has steadily decreased globally during the last decade, it has been more frequently diagnosed among young individuals in recent years [7, 8]. Similar trends have been reported in Sub-Saharan Africa (SSA), where it affects a relatively younger population [6]. These alarming trends merit particular attention as they may have a more significant economic, health, societal effect given the higher number of productive years of life at risk [9].

The global incidence of ACS among young individuals is difficult to establish due to its atypical presentation and variability in how it is reported in different healthcare systems [10]. Several large multicenter observational studies conducted outside the African continent have reported an incidence between 4–10% [8, 11–16]. Reports from Northern America, Europe, some parts of Asia have reported unique characteristics with distinct risk factors [14, 15, 17–20], clinical presentation [11], angiographic severity [16, 21–23], clinical outcomes [12, 14, 24] when compared to older patients. Nevertheless, the findings are not uniform and vary significantly across different cohorts. Despite the epidemiological transition, healthcare systems in Africa are traditionally geared towards addressing infectious diseases. Data on ACS is limited in the African continent and more so poorly reported in the young [6]. To date, no study has reviewed the spectrum of ACS among young individuals in Sub-Saharan Africa (SSA). Recent national reports indicate high CVD mortality in Kenya with more than 50% occurring prematurely [25].

In light of this background, we aimed to describe the clinical spectrum of ACS among young individuals in Kenya and elucidate prevalent risk factors. Insights gained from this report will identify prognostic characteristics and generate intervention targets.

## Methods

### Study design and settings

This was a single-center retrospective cohort study conducted at the Aga Khan University Hospital, Nairobi (AKUH, N). The AKUH, N is one of the largest private hospitals in the country with a current inpatient capacity of 289 beds. The Aga Khan University hospital Nairobi is the first hospital in Kenya to be accredited by the Joint Commission International (JCI) accreditation, USA and the only hospital in the region accredited for the management of heart attacks by JCI. This accreditation testifies commitment to provide quality patient care guided by patient safety standards aimed at good clinical outcomes. The inpatient cardiology section includes a 24-hour operational catherization laboratory and a 6 bed Coronary Care Unit (CCU) able to provide Level III [26] cardiovascular care with a 24-hour coverage by on call cardiology fellow and an interventional cardiologist. A 24 h back up cardiothoracic surgeon is available when the need arises. Patients admitted to the CCU receive a 1:1 nurse to patient care. The Cardiology section of the AKUHN is registered with Cardiovascular Data Registry (NCDR- CathPCI). The CathPCI is the American College of Cardiology (ACC) suite of data registries which help hospital’s improve quality of cardiovascular care they provide committed to ensuring evidence-based practice while improving patient outcomes. This powerful tool captures adherence to ACC/AHA clinical practice guideline recommendations, procedure performance standards and appropriate use criteria (AUC) for coronary revascularization. Data into the NCDR- CathPCI is entered by a designated research nurse and then verified by the interventional cardiologist for accuracy and completeness on a case to case basis. Variables (demographics, laboratory parameters, patient specific treatments, reperfusion strategies and in-hospital outcomes) collected are recommended by the ACC- NCDR- CathPCI [27].

### Study population and sample size determination

**Fig. 1:**
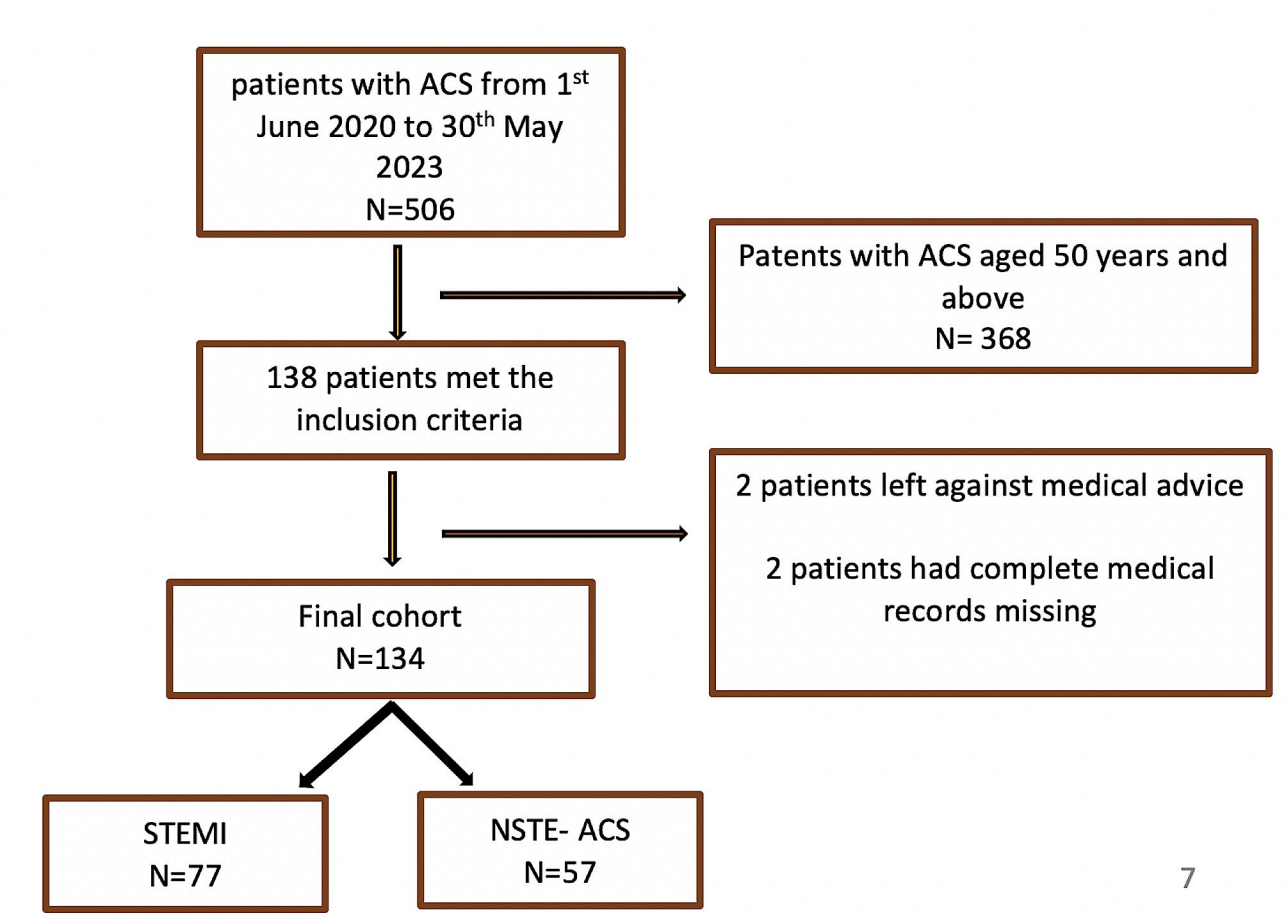
Flow diagram illustrating the selection of the study population included in the study

The current study involved extracting data from CathPCI registry of all the young individuals who presented with ACS from 1st June 2020 to 31st May 2023. We defined “young” with the age below 50 years. There has not been a universal consensus definition of young in regard to ACS but often considered reasonable and justified by various other studies for individuals below 50 years of age [9, 28–30]. All patients aged 50 years or below presenting with Acute Coronary Syndrome (ACS) were included in the study. Patients with complete missing medical records, transferred to other facilities and those leaving against medical advice were excluded from the study. The diagnosis of ACS was based on patients’ clinical signs and symptoms, electrocardiographic changes and elevation of cardiac - biomarkers [31]. Patients were grouped into two categories: (i) ST-elevation myocardial infarction (STEMI) and (ii)non-ST elevation Acute coronary Syndrome (NSTE- ACS) (Fig. 1). Evidence-based assessment, risk stratification and therapeutic strategy are followed by the institution as indicated in updated guidelines by American college of cardiology/ American Heart Association (ACC/AHA) [32, 33] Electrocardiograph (ECG) changes were documented according to the territory involved [34]. Experienced interventional cardiologists determined the cause of ACS according to previous validated methods [35, 36]. The angiographic characteristics were grouped into atherosclerotic [36], thrombotic [36] and spontaneous coronary artery dissection [35, 37]. Significant CAD was defined by invasive coronary angiography as a narrowing of the internal diameter > 50% stenosis of the left main stem and > 70% stenosis in a major coronary vessel [38]. Percentage diameter less than the mentioned above was characterized as non-obstructive coronary artery disease [39]. 2D echocardiography parameters prior to discharge were documented and confirmed by attending cardiologists. Left Ventricular function was classified as per the recommendations of the American Society of Echocardiography (ASE) [40].Patients were followed up to hospital discharge and grouped as alive or dead. The admission and CathPCI NCDR were used to identify patients who met the inclusion criteria.

### Data management

All available data in paper-based and electronic format were obtained from the institutional medical records department. Data extracted included demographic characteristics, past medical history, underlying comorbid illness, symptoms on presentation, duration of symptoms, ACS diagnosis, angiographic findings and in- hospital outcome. Data were extracted by the primary investigator and randomly verified by a supervising faculty for accuracy and completeness. Data was collected in the MS Excel 2020.

The Study protocol was presented at the section level and department level before obtained ethical clearance from the Aga Khan university - Institution Scientific and Ethic Review Committee (ISERC). The study accepted by the Scientific and Ethic Review Committee (ISERC) of the Aga Khan University, Hospital Nairobi. Reference number Ref: AKU/2023/ISERC-65 (vl).

### Data analysis

Data analysis was performed using R version 4.1.3 (2022-03-10). Categorical variables were reported as frequencies and percentages and compared with chi- squared or Fisher’s exact tests. Continuous variables were summarized descriptively as means or medians with the corresponding measures of variability and compared with t-tests or Mann-Whitney U tests. Statistical significance was set at P- Value < 0.05.

## Results

A total of 506 patients presented with ACS during the study period, of which 138 patients met the inclusion criteria. 4 patients were excluded from the final analysis as seen in Fig. 1 above. The incidence of ACS among young individuals presenting to our institution was 27.2% (95% CI:22.8-30.5%).

The median age of our cohort was 46.5 years (IQR 41.0–50.0). Our cohort was male (*n* = 107,79.9%) and African predominant (*n* = 82, 61.2%) as seen in Table 1 below. The median BMI of our cohort was 29.6 Kg/m^2^ (IQR 26.0–32.0) of which only a fraction had a normal BMI (*n* = 15, 14.9%). The youngest patient was 31 years old.

**Table 1:** Basic demographic profile of the cohort

Variable	Overall, *N* = 134^1^	STEMI, *N* = 77^1^	NSTEMI, *N* = 57^1^	p-value^2^
Age in years, Median (IQR)	46.5 (41.0–50.0)	46.0 (41.0–50.0)	47.0 (42.0–50.0)	0.79
Categories of age-group, n (%) < 40 41–45 45–50	31(23.1) 30(22.4) 73(54.5)	18(23.4) 16(20.8) 43(55.8)	13(22.8) 14(24.6) 30(52.6)	0.87
Ethnicity, n (%) African South Asian Other	82(61.2) 40(29.9) 12(9.0)	48(62.3) 25(32.5) 4(5.2)	34(59.6) 15(26.3) 8(4.0)	0.19
Gender, n (%) Female Male	27(20.1) 107(79.9)	10(13.0) 67(87.0)	17(29.8) 40(70.2)	0.016
Body Mass Index (BMI), Median (IQR)	29.6 (26.0–32.0)	28.3 (25.7–32.0)	30.0 (26.9–32.3)	0.23
Categories of BMI, n (%) Normal (18.5–24.9 Kg/m2) Overweight (25-29.9 Kg/m2) Obese (> 30 Kg/m2)	15(14.9) 39(38.6) 47(46.5)	9(15.8) 25(43.9) 23(40.4)	6(13.6) 14(31.8) 24(54.5)	0.39

^1^Median (IQR) or Frequency (%) ^2^Wilcoxon rank sum test; Pearson’s Chi-squared test; Fisher’s exact test. NSTEMI: Non- ST Elevation Myocardial Infarction, STEMI: ST- Elevation Myocardial Infarction, BMI: Body Mass Index.

More than one comorbid condition per patient was recorded when present. The most common comorbid condition amongst our cohort was hypertension (*n* = 101,75.4%). Approximately half of the study population were smokers(*n* = 69,51.5%), had a family history of premature ASCVD (*n* = 72,53.7%) and were on cholesterol-lowering medication (*n* = 68, 50.7%) prior to presentation. Among diabetic individuals (*n* = 58, 43.2%) the majority had suboptimal control with HbA1c above 7% (*n* = 33, 64.7%) as seen in Table 2 below.

**Table 2:** Underlying risk and comorbid condition

	Overall, *N* = 134^1^	STEMI, *N* = 77^1^	NSTEMI, *N* = 57^1^	p-value^2^
HTN, n (%)	101 (75.4)	56 (72.7)	45 (78.9)	0.41
DM, n (%)	58 (43.2)	33 (42.9)	19 (33.3)	0.26
Both DM and HTN n (%)	44 (32.8)	28 (36.4)	16 (28.1)	0.31
HBA1C, Median (IQR)	9.0 (6.9–10.8)	9.0 (6.9–10.6)	8.0 (7.0–10.5)	0.95
**Categories of HBA1C** n (%) **< 6.5%** **6.5-7%** **> 7%**	7(13.7) 11(21.6) 33(64.7)	5(15.6) 6(18.8) 21(65.6)	2(10.5) 5(26.3) 12(63.2)	0.83
**Therapy among DM**, n (%) Non- Pharmacotherapy Oral Insulin Oral and insulin	5(8.6) 31(53.4) 14(24.1) 8(13.8)	5(12.8) 21(53.8) 7(17.9) 6(15.4)	0(0) 10(52.6) 7(36.8) 2(10.5)	0.24
**Smoking**	69 (51.5)	43(55.8)	26(45.6)	0.90
**On Cholesterol lowering medication prior presentation**, n (%)	68 (50.7)	33(42.9)	35(61.4)	0.13
**Previous MI**, n (%)	16 [11]	8(10.4)	8 [14]	0.52
**Family history of Premature ASCVD**, n (%)	72(53.7)	43(55.8)	29(50.8)	0.33
**Other Comorbid Conditions, n** (%) Hypothyroidism Hyperthyroidism CKD and ESRD HIV Connective tissue disease Asthma Polycythemia	14(10.4) 3(2.2) 8(5.9) 3(2.2) 12(8.9) 3(2.2) 6(4.5)	6(7.8%) 3(3.9) 2(2.6) 3(3.9) 5(6.5) 1(1.3) 5(6.5)	8(14.1) 0(0.0) 6(10.5) 0(0) 7(12.3) 2(3.6) 1(1.8)	0.33

^1^n (%); Median (IQR), ^2^Wilcoxon rank sum test; Pearson’s Chi-squared test; Fisher’s exact test. HBA1C: Glycated Hemoglobin, DM: Diabetes Mellitus, HTN: Systemic arterial hypertension, MI: Myocardial Infarction, CAD: Coronary Artery Disease, ASCVD: Atherosclerotic Cardiovascular Disease CKD: Chronic Kidney Disease, ESRD: Ends stage renal disease, HIV: Human immunodeficiency Virus.

The most common presentation was chest pain (*n* = 109,81.3%). The median duration of chest pain for patients with STEMI was (6.1 h, IQR 2–8) lower than those with NSTE- ACS (12 h, IQR 3.8–24). The Majority had no evidence of heart failure (*n* = 94, 70.1%) on presentation. Anterior- Septal was the most common ECG territory affected (*n* = 84, 62.6%) as seen in Table 3 below.

**Table 3:** Clinical presentation on the cohort

Variable	Overall, *N* = 134^1^	STEMI, *N* = 77^1^	NSTEMI, *N* = 57^1^	p-value^2^
**Presenting Complain** Chest Pain, n (%) Difficulty in breathing, n (%) Epigastric pain, n (%) Palpitations, n (%) Syncope, n (%) V- Fib Arrest, n (%)	109(81.3) 2(1.5) 12(9.0) 6(4.5) 3(2.2) 2(1.5)	64(83.1) 0(0.0) 4(5.2) 5(6.5) 2(2.6) 0(0)	45(78.9) 2(3.5) 8(14.0) 1(1.8) 1(1.8) 2(3.5)	0.10
Duration of Chest Pain (IQR)	10.0(2.0–16.0)	6.1 [2–8]	12.3(3.8–24)	0.019
**Killip Classification** Killip 1, n (%) Killip > 2, n (%)	94 (70.1%) 50 (42.4%)	53(68.8) 24(31.1)	41(71.9) 26(45.6)	0.61
**ECG Involvement** Anterior- septal, n (%) Inferior, n (%) Lateral, n (%) Posterior, n (%) Complete heart block,n (%)	84 (62.6%) 36 (26.8%) 11(8.2%) 2 (1.5%) 1 (0.7%)	50(64.9) 19(24.7) 6(7.8) 1(1.3) 1(1.3)	34(59.6) 17(29.8) 5(8.8) 1(1.8) 0(0.)	0.22

^1^n (%); Median (IQR), ^2^Wilcoxon rank sum test; Pearson’s Chi-squared test; Fisher’s exact test. V- Fib: Ventricular Fibrillation

Significant coronary stenosis was identified in more than 50% of individuals (*n* = 75,56%), single vessel disease was the main coronary finding(*n* = 60,80%) with a predilection of the Left anterior descending artery(*n* = 42,62.6%). Atherosclerosis(*n* = 41,54.6%) of the coronary artery was the main cause of ACS in our cohort as seen in Table 4 below.

**Table 4:** Comparison of angiographic parameters of the cohort

**Variable**	n	**Overall**, *N* = 134^1^	**STEMI**, *N* = 77^1^	**NSTE-ACS**, *N* = 57^1^	**p-value** ^2^
**Angiographic Findings** Non - Obstructive CAD Significant CAD	132	57 (43.2%) 75(56.8%)	25 (32.5%) 52(67.5%)	32 (56.1%) 23 (40.4%)	0.002
**Obstructive CAD** Single Vessel Two Vessel Triple Vessel	75	60 (80%) 10(13.3%) 5(6.7%)	45 (86.5%) 6(11.5%) 1(1.9%)	15 (65.2%) 4 (17.3%) 4 (17.3%)	< 0.001
**Culprit Vessel** **LAD** **LCX** **RCA**	75	47(62.6%) 6(8.1%) 22(29.3%)	31(59.6%) 4(7.6%) 17(32.7%)	16(69.6%) 2(8.7%) 5(21.7%)	0.078
**Etiology of Culprit** Atherosclerotic Thrombotic SCAD	75	41(54.6%) 30(40%) 4(5.4%)	21(40.4%) 28(53.8%) 3(5.7%)	20(86.9%) 2(8.7%) 1(4.34%)	0.05
**Coronary Ectasias**	132	9 (6.7%)	6 (7.8%)	3 (5.3%)	0.73

^1^n (%); Median (IQR), ^2^Wilcoxon rank sum test; Pearson’s Chi-squared test; Fisher’s exact test. LAD: Left Anterior Descending Artery, LCX: Left Circumflex Artery, RCA: Right Coronary Artery, SCAD: Spontaneous Coronary Artery Dissection.

Patients with STEMI had a lower mean HDL when compared with patients with NSTEMI. Other Laboratory parameters are illustrated in Table 5 below.

**Table 5:** Laboratory Parameters of the cohort

	Overall, *N* = 134	STEMI, *N* = 77	NSTEMI, *N* = 57	P-value^1^
TC, Mean (SD)	4.9 (1.3)	4.7 (1.1)	5.1 (1.5)	0.14
HDL, Mean (SD)	1.0 (0.4)	1.0 (0.2)	1.1 (0.5)	0.029
LDL, Mean (SD)	3.6 (1.4)	3.5 (1.1)	3.6 (1.7)	0.60
TG, Mean (SD)	1.9 (1.0)	1.9 (0.8)	2.0 (1.3)	0.41
Creatinine, Mean (SD)	105.0 (88.5)	98.7 (33.4)	113.3 (129.2)	0.42
HB, Mean (SD)	14.7 (2.0)	15.0 (2.0)	14.3 (2.1)	0.072
PLT, Mean (SD)	272.5 (91.3)	280.8 (96.4)	261.2 (83.4)	0.23
CRP, Mean (SD)	44.2 (55.5)	41.3 (58.7)	48.1 (52.0)	0.64

1: Welch Two Sample t- test TC (mmol/l): Total Cholesterol, HDL (mmol/l): high Density Lipoprotein, LDL( mmol/l): Low Density Lipoprotein, TG(mmol/l): Triglyceride, HB(mg/dl): Hemoglobin, PLT*109/L: Platelet, CRP (mmol/l): C- Reactive Protein.

Table 6 Below illustrates the reperfusion strategy among patients with STEMI. More than half (*n* = 52, 67.5%) underwent coronary intervention as illustrated below.

**Table 6:** Intervention among STEMI - Patients

Intervention	STEMI, *N* = 77^1^
Thrombolysis	10 (12.9%)
Primary PCI	34 (65.9%)
Rescue PCI	9 (11.6%)
Primary PCI + Staged PCI (PCI of non-IRA)	4 (5.2%)
Aspiration thrombectomy + Primary PCI	3 (3.9%)
POBA	2 (2.6%)
Coronary Angiography without intervention	24 (35.1%)
Coronary Angiography - Post thrombolysis, without intervention	1 (1.3%)
Eptifibatide Infusion	16(20.8%)
DAPT therapy on discharge amongst patients with significant CAD (*n* = 50) Aspirin + Clopidogrel Aspirin + Ticagrelor Aspirin + Prasugrel Triple therapy with NOAC Triple Therapy with Warfarin	11 (22%) 33 (66%) 6 (12%) 9 (11.6%) 1 (1.2%)

1: Frequency (%), POBA: Percutaneous Old Balloon Angioplasty, PCI: Percutaneous Coronary Intervention, DAPT: Dual Anti- Platelet Therapy, CAD: Coronary Artery Disease, NOAC: Novel Oral Anti- Coagulation, IRA: Infarct Related Artery.

Table 7 below illustrates the type of Intervention and strategy performed among patients with NSTE - ACS. The most common strategy was intervention within the first 24 h (*n* = 40, 70.2%). More than half of the population did not require any form of revascularization (*n* = 32, 56.1%).

**Table 7:** Risk stratification and revascularization strategy and Interventions done for patients with NSTE- ACS

Intervention and Strategy	NSTE-ACS, *N* = 57^1^
TIMI - Risk for NSTE ACS 0–2 (low) 3–4 (intermediate) 5–7 (High)	11(19.2%) 20(35.1%) 26(45.6%)
Type of Intervention	
Immediate Invasive (< 2 h), **n (%)**	24 (42.1%)
Early Invasive (< 24 h), **n (%)**	16 (28.1%)
Invasive Strategy (< 72 h), **n (%)**	15(26.3%)
Medical Management, **n (%)**	2 (3.5%)
Coronary angiography - without intervention	32 (56.1%)
Coronary angiography + PCI	12 (21.1%)
Coronary angiography - referred for CABG	6 (10.5%)
Coronary angiography + PCI in 2vessel	5 (8.8%)
No angiography - Medical management	2 (3.5%)
**Eptifibatide Infusion**	2(3.5%)
DAPT therapy on discharge amongst patients with significant CAD (*n* = 23) Aspirin + Clopidogrel Aspirin + Ticagrelor Aspirin + Prasugrel Triple therapy with NOAC Triple Therapy with Warfarin	15 (65.2%) 6 (26.0%) 2 (8.6%) 2 (3.7%) 1 (1.9%)

1: Frequency (%), TIMI: Thrombolysis in Myocardial Infarction, CABG: Coronary Artery Bypass Graft, POBA: Percutaneous Old Balloon Angioplasty, PCI: Percutaneous Coronary Intervention, DAPT: Dual Anti- Platelet Therapy, CAD: Coronary Artery Disease, NOAC: Novel Oral Anti- Coagulation, IRA: Infarct Related Artery,

The Mean Left Ventricular Ejection fraction (LVEF) was 46.0 (± 12.4). Statistical significance was noted (P Value 0.05) when the mean LVEF of patients with STEMI and NSTE - ACS were compared as seen below in Table 8.

**Table 8:** Echocardiographic parameters prior to discharge

	N	Overall, *N* = 134^1^	NSTE-ACS, *N* = 57^1^	STEMI, *N* = 77^1^	p-value^2^
Mean LVEF	113	46.0 (± 12.4)	48.6 (± 11.5)	44.2 (± 12.8)	0.05
**Severity of LV Dysfunction**					0.17
Normal		47 (41.6%)	24 (51.1%)	23 (34.8%)	
Mild		29 (25.7%)	13 (27.7%)	16 (24.2%)	
Moderate		22 (19.5%)	6 (12.8%)	16 (24.2%)	
Severe		15 (13.3%)	4 (8.5%)	11 (16.7%)	
**RWMA**					0.26
Absent		53 (46.9%)	25 (53.2%)	28 (42.4%)	
Present		60 (53.1%)	22 (46.8%)	38 (57.6%)	
**LVTHROMBUS**					0.36
Absent		102 (90.3%)	44 (93.6%)	58 (87.9%)	
Present		11 (9.7%)	3 (6.4%)	8 (12.1%)	

1: Median (IQR) or Frequency, 2: Wilcoxon rank sum tests, Pearson’s chi- squared; Fisher exact. LVEF : Left Ventricular Ejection Fraction, LV : Left Ventricle.

The median length of stay was 3 days (IQR 3.0–5.0). Prolonged hospital stay was noted in individuals with STEMI. The in-hospital mortality rate amongst our cohort was 1.5% as seen in Table 9 below.

**Table 9:** Outcome at hospital discharge of our cohort

Variable		Overall, *N* = 134^1^	NSTE-ACS, *N* = 57^1^	STEMI, *N* = 77^1^	p-value^2^
**Length of stay**	134	3.0 (3.0, 5.0)	3.0 (2.0, 4.0)	4.0 (3.0, 5.0)	0.003
**Outcome** Alive Dead	134	132 (98.5%) 2 (1.5%)	56 (98.2%) 1 (1.8%)	76 (98.7%) 1 (1.3%)	0.004

1: Median (IQR), n (%); 2 Wilcoxon rank sum tests; Pearson’s Chi-squared test

**Fig. 2:**
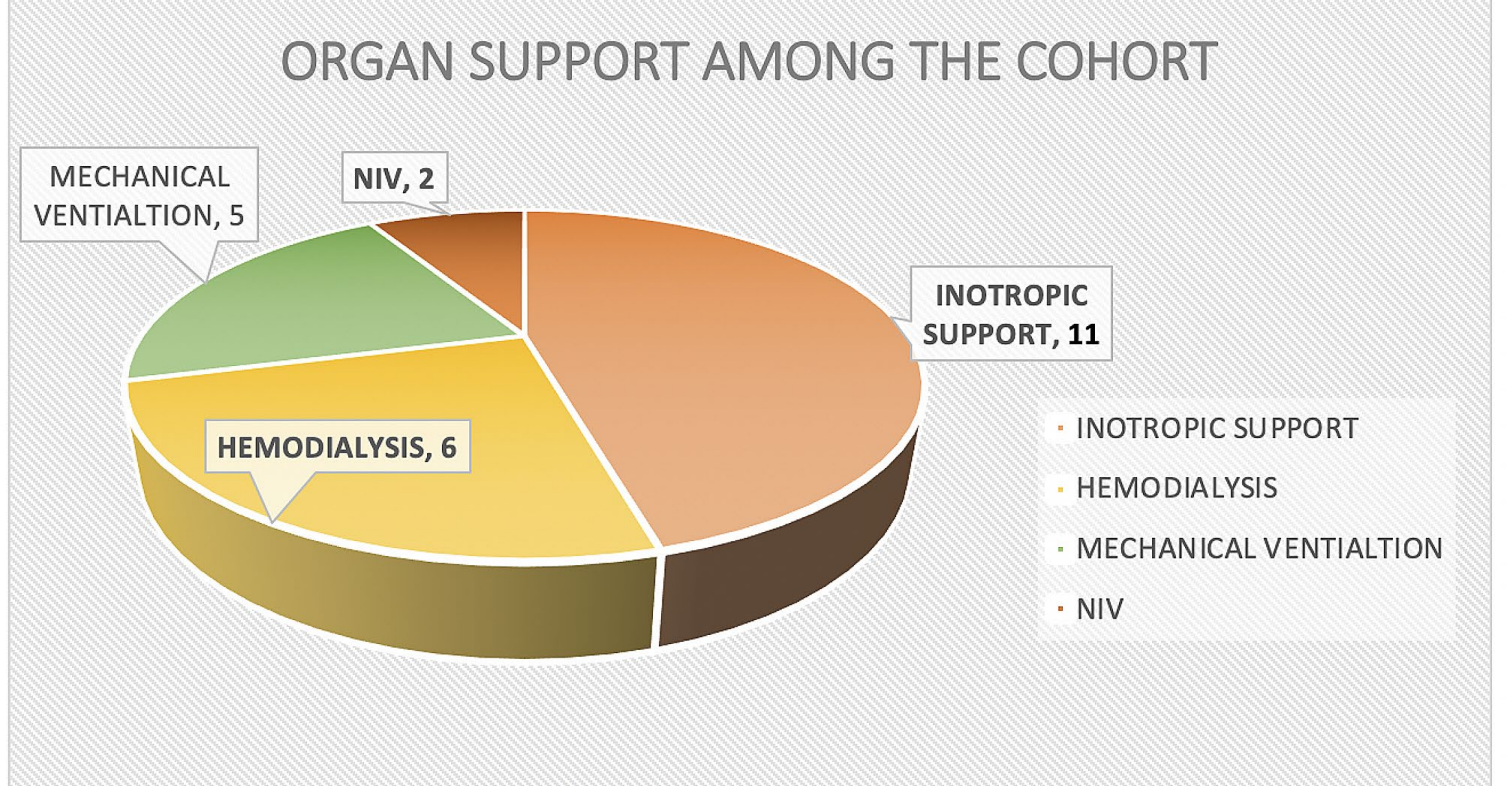
Organ support amongst our cohort. NIV: Non-Invasive Ventilation

Figure 2 below illustrates the organ support needed amongst our cohort. Inotropic support was the most frequently used organ support.

## Discussion

To the best of our knowledge, we present one of the first studies to report on ACS among young individuals in the African continent. Findings from this study indicate that approximately 25% of patients hospitalized with ACS at our center were less than or equal to 50 years of age. This confirms a higher proportion compared to age- matched registry studies done in the Western world, which reported an incidence between 4 and 10% [8, 11–16]. We hypothesize the higher proportion to the increased prevalence of cardiovascular risk factors in our population, namely; diabetes mellitus, hypertension, obesity, and cigarette smoking. These factors are considered silent preventable killers and the main reasons for premature CAD globally and in SSA. Registry studies have documented the aforementioned to be highly prevalent in the East African region [41–43]. These observations support the present-day emphasis on controlling cardiovascular risk factors and quitting cigarette smoking to prevent adult coronary artery disease. Additionally, the presence of ASCVD risk enhancers [44] and presence of at risk population [45] in our cohort could account for a higher proportion of ACS among young individuals.

Despite differences in geographical and population dynamics. The demographic, clinical profile, risk, and sex distribution of our cohort are similar to studies conducted in Northern America, Asia, and Europe [11, 12, 46]. Our study population was male-predominant and followed a pattern observed in previous studies. Nonetheless, 20% of our patients were females; representing a higher percentage than observed by other comparable studies [11, 12]. Young women with ACS comprise an especially interesting group given the protective effect of estrogen. This paradox has been associated with: Psychosocial [17], abnormal menstrual cycle [18] and oral contraceptive use [47]. More importantly, few studies [30] have suggested sex- specific influence on ACS outcomes with young women having a higher 30-day mortality. The scope of our study and the small sample size limited us to explore this association in our cohort.

The study showed that majority of the individuals presented with classical chest pain as their main symptom. Young individuals are less likely to present with typical chest pain, a common symptomology of ACS [11]. Instead, they present with atypical symptoms such as awareness of heartbeat, nausea, fatigue, and dizziness [11]. These atypical presentations might account for the lower threshold of detection. Findings from our study describe a higher rate of STEMI as observed in various other age matched cohorts [12, 20, 48].

Cigarette smoking has been identified as the primary modifiable factor among young individuals in various populations [8, 14, 15] with a higher rate noted in the very young and linked with increased readmission rates, need for revascularization, and poor 5-year survival [15, 16]. Our results are parallel and depict similar trends with more than half of our cohort involved in cigarette smoking. Although dyslipidemia is an important risk factor for young, there seems to be little difference when compared to older patients. Several studies have reported increased triglyceride [19] and low HDL [19, 20] as the main factors associated with ACS in the young. Our study findings are comparable to reports published globally. Of note, approximately half of our cohort was already on cholesterol- lowering medications and yet presented with an acute coronary event suggesting failed appropriate primary or secondary prevention of ASCVD. This raises concern about either compliance among patients or incorrect doses of statins prescribed by healthcare workers, a major observation noted in clinical practice. Due to the study’s retrospective design, both mentioned parameters were not objectively assessed. Poor compliance and under-dosing of statin therapy predispose an individual to heightened cardiovascular risk, progression of coronary artery disease and increased health care costs [49]. These factors have been observed across patient subgroups (younger individuals) and by the intensity of statin therapy (high intensity) [50]. This analysis is therefore timely and creates a considerable opportunity for improvement in the primary prevention of ASCVD [50]. Furthermore, obesity appears to be an independent risk factor for premature coronary atherosclerosis especially, in young males [51], our cohort is a mere reflection of the at-risk population. Recent reports have indicated a better index [52] in predicting premature CAD than BMI and waist circumference and could be area of further research in different ethnic cohorts. Additionally, family history of premature ASCVD was a strong elucidated risk in our study population with reports validating it is a strong surrogate of premature ASCVD [53]. Polymorphism in novel genes among young individuals has been linked with the progression of atherosclerosis and CAD [52] with scientific evidence to support Lipoprotein a (LPa) measurement in identifying young individuals at heightened risk of premature ASCVD [54, 55].

Young Individuals presenting with ACS have a higher incidence of normal coronary arteries [23, 56] with only mild luminal narrowing. Our study findings contrast to what has been published globally and rather depict a higher frequency of angiographically significant coronary artery disease. The scope of the current study was beyond explaining the difference. A Strong family history of premature ASCVD and a higher prevalence of traditional and modifiable atherothrombotic risks may account for the disparity. The findings of predominately single vessel disease [12, 24] and predilection of Left Anterior Descending Artery (LAD) [16, 21] are in accord with previous studies. COVID − 19 myocarditis and vasospastic angina both mimic ACS in clinical presentation and might be accountable for our cohorts non - obstructive coronary artery disease.

The in-hospital mortality amongst our cohort was 1.5%. Due to the lower mortality rate, we couldn’t elucidate factors associated. Similar mortality rates have been documented in western series ranging between 0 to 4% [15, 16, 24]. The low in- hospital morality can be addressed in two folds. Firstly, Young individuals have favorable outcomes primarily due to the less advanced atherosclerosis and exhibit enhanced response to medical therapy. Additionally, besides, better physiological reserve and better collateral circulation better; the AKUHN has also heavily invested in care for patients with ACS. The AKUHN has been at the forefront of quality cardiac care for the past 10 years within the region.

Findings of the current study, add to the already existing knowledge. Data on the incidence of ACS especially among young adults are lacking and poorly documented in the Sub-Saharan region. This analysis presents a forward step in exploring the rising incidence of ACS among young individuals. To fully comprehend the effect of this syndrome; in detail, multi-center, case- control studies are needed with an exploration of contextual risk factors. The AKUHN follows protocols and guidelines from the European Society of Cardiology (ESC) and the American College of Cardiology (ACC) and there have been no major changes in the core components of the management of ACS from the recently ended ESC congress.

## Limitations

The main purpose of this study was to understand the magnitude of ACS among young individuals in Kenya as well as to obtain contextual risks associated. Nonetheless, it has to be interpreted in light of some limitations. First, its retrospective design; which contained discharge level records and as such is inclined to missing data that hindered us exploring various variables such risk factors, biophysical profiles, several comorbidities, vitals on presentation, education level, marital status and ethnicity. However, We tried our best to extract as much as we could from medical records and databases available for consistent statistical analysis. Another limitation is the lack of a control group of older patients, nevertheless, this was never the primary objective of the study. Since global data on ACS among young is abundant we used it for reference and discussion. Despite the study setting severing more than 25% of heart attacks in Kenya, this was a single-center study conducted in a private hospital; thus, results cannot be generalized nationwide. An ACS diagnosis in young individuals could be challenging since myocarditis can mimic similar symptomatology leading to selection bias. Our data did not have data on the use of substances such as sympathomimetic amines [57] and khat consumption [58] which have been found to correlate with ACS, especially at a younger age. Finally, there is no universal definition of young patients, commonly accepted age range is below 50 years. This may be a confounding factor when comparing literature across various cohorts.

## Future directions

The epidemiological profile of patients with Acute Myocardial Infarction (AMI) is changing and is now no longer the disease of the elderly requiring health care workers to become effective in diagnosing ACS in all patient population, regardless of age and sex. Moving Forward, sustained advocacy and investment in primordial and primary prevention of ASCVD is paramount and remains the beast weapon in resource limited setting. The African continent has witnessed a rapid surge in cardiovascular risk factors, especially in a much younger population. We feel, the region is unprepared for the growing burden with evident deficiencies in health care infrastructure. Findings from the studies should attract the community’s attention to seek a healthier life style and better control cardiovascular risk factors. The real time epidemiological transition calls out for a nation- wide implementation in formulating suitable strategies in identifying young individuals at heightened risk for death from CAD. These efforts are essential to offset the enormous costs associated with the care of premature ACS.

## Conclusion

Young patients in Kenya contribute to a relatively large proportion of patients presenting with ACS. The majority were men, more than half of the cohort were cigarette smokers and overweight with a fraction having underlying diabetes mellitus and hypertension. Modifiable risk factors are targets of intervention for healthcare workers.

### Electronic supplementary material

Below is the link to the electronic supplementary material.

Supplementary Material 1

Supplementary Material 2

## Data Availability

The datasets used and/or analyzed during the current study are available from the corresponding author on reasonable request.

## Abbreviations

ACS: Acute Coronary Syndrome
SSA: Sub- Saharan Africa
STEMI: ST- Elevation Myocardial Infarction
NSTEMI: Non- ST Elevation Myocardial infarction
LMICs: Low Middle Income Countries
CVD: Cardiovascular Disease
NCD: • Non-Communicable Diseases
• CAD: Coronary Artery Disease
AKUH, N: Aga Khan University Hospital Nairobi
JCI: Joint Commission International
NCDR: National Cardiovascular Data Registry
ACC: American College of Cardiology
CCU: Coronary Care Unit
AHA: American Heart Association
PCI: Percutaneous Intervention
ASE: American Society of Echocardiography
LVEF: Left Ventricular Ejection Fraction
PCI: Percutaneous Coronary Intervention
