# Supplementary material for: Acute coronary syndrome patterns in the Young: risk factor profile and in-hospital outcomes in a tertiary referral hospital in Kenya
Source: BMC Cardiovasc Disord. 2024 Apr 3;24:192. doi: 10.1186/s12872-024-03832-z (PMC10988889; doi:10.1186/s12872-024-03832-z)
Supplement: Supplementary file 1 — Supplementary Material 1 [file 12872_2024_3832_MOESM1_ESM.docx]

rm(list=ls())

library(tidyverse)

library(xtable)

library(readxl)

library("writexl")

library(readr)

library(finalfit)

library(officer)

library(summarytools)

library(gtsummary)

setwd("C:\\Users\\James\\OneDrive\\Documents\\residents\\nadeem\\ICUdata")

excel_sheets('FINAL - FELLOW DATA.xlsx')

theme_gtsummary_journal(journal = "jama")

theme_gtsummary_compact()

sect_properties <- prop_section(

page_size = page_size(orient = "portrait",

width = 10.0, height = 11.7),

type = "continuous",

page_margins = page_mar()

)

#################Importing the dataset############################

dataset=read_excel("Final data.xlsx",sheet = "Sheet1")

#View(dataset)

names(dataset)

dataset$agegrp[dataset$AGE>=22 & dataset$AGE<=40]<-1

dataset$agegrp[dataset$AGE>=41 & dataset$AGE<=45]<-2

dataset$agegrp[dataset$AGE>=46 & dataset$AGE<=50]<-3

dataset$agegrp=factor(dataset$agegrp,levels=c(1:3),label=c("<40 ","41- 45","45 -50"))

dataset$BMIVATGORY[dataset$BMI>=13.26 & dataset$BMI<=18.97]<-1

dataset$BMIVATGORY[dataset$BMI>=19.0 & dataset$BMI<=24.98]<-2

dataset$BMIVATGORY[dataset$BMI>=25.0 & dataset$BMI<=29.9]<-3

dataset$BMIVATGORY[dataset$BMI>=30.0 & dataset$BMI<=58.0]<-4

dataset$BMIVATGORY=factor(dataset$BMIVATGORY,levels = c(1:4),

labels = c("<18.5","19-24.9","25-29.9",">30"))

names(dataset)[names(dataset)=="EMEREGNCY/ TRASNFERED FROM WARD"]<-"source"

dataset|>

dplyr::select(AGE,agegrp,ETHNICITY,SEX,BMI,BMIVATGORY,source,OUTCOME)|>

tbl_summary(by=OUTCOME,

missing='no',

digits = list(all_continuous() ~ 1,

all_categorical()~c(0,1)),

label = c(AGE~"Age in years",

agegrp~"Categories of age-group",

ETHNICITY~"Ethinicity",

SEX~"Gender",

source~"EMEREGNCY/ TRASNFERED FROM WARD",

BMI~"Body Mass Index (BMI)",

BMIVATGORY~"Categories of BMI"))|>

modify_header(label ~ "**Variable**")|>

modify_caption("**Table 1: Baseline Demographic Characteristics**")|>

bold_labels()|>

add_overall()|>

add_p(pvalue_fun = ~ style_pvalue(.x, digits = 2))|>

modify_header(label ~ "**Variable**") %>%

modify_spanning_header(c("stat_1", "stat_2") ~ "**Outcome**") %>%

modify_footnote(

all_stat_cols() ~ "Median (IQR) or Frequency (%)") %>%

as_flex_table()|>

flextable::save_as_docx(demo,path = ("C:\\Users\\James\\OneDrive\\Documents\\residents\\nadeem\\ICUdata\\Table 1.docx"), pr_section = sect_properties)

####

dataset$hba1cgrp[dataset$HBA1C>=5.64 & dataset$HBA1C<6.5]<-1

dataset$hba1cgrp[dataset$HBA1C>=6.5 & dataset$HBA1C<=7.0]<-2

dataset$hba1cgrp[dataset$HBA1C> 7.0 & dataset$HBA1C<=14.6]<-3

dataset$hba1cgrp=factor(dataset$hba1cgrp,levels = c(1:3),labels = c("< 6.5%","6.5-7%","> 7%"))

names(dataset)[names(dataset)=="CKD/ESRD"]<-"CKD"

names(dataset)[names(dataset)=="LIVER CIRHOSIS"]<-"LIVER"

names(dataset)[names(dataset)=="HEART FAILURE"]<-"HEART"

dataset$COMORBID=factor(dataset$COMORBID,levels = c(0:4),

labels = c("None",

"One",

"Two",

"Three",

"Four Plus"))

dataset|>

dplyr::select(COMORBID,HTN,DM,CKD,HIV,LIVER,EPILEPSY,CAD,HYPOTHYRODISM,HEART,OUTCOME)|>

tbl_summary(by=OUTCOME,

missing='no',

digits = list(all_continuous() ~ 1,

all_categorical()~c(0,1)),

label = c(HTN~"Presence of HTN",

DM~"Presence of DM",

CKD~"Presence of CKD",

HIV~"Presence of HIV",

LIVER~"Presence of Liver Cirhosis",

EPILEPSY~"Presence of Epilepsy",

CAD~"Presence of CAD",

HYPOTHYRODISM~"Presence of HYPOTHYRODISM" ,

HEART~"Presence of heart failure"))|>

modify_header(label ~ "**Variable**")|>

modify_caption("**Table 2: Risk Factors for ACS**")|>

bold_labels()|>

add_overall()|>

add_p(pvalue_fun = ~ style_pvalue(.x, digits = 2))|>

modify_header(label ~ "**Variable**") %>%

modify_spanning_header(c("stat_1", "stat_2") ~ "**Outcome**") %>%

modify_footnote(

all_stat_cols() ~ "Frequency (%)") %>%

as_flex_table()|>

flextable::save_as_docx(demo,path = ("C:\\Users\\James\\OneDrive\\Documents\\residents\\nadeem\\ICUdata\\Table 2.docx"), pr_section = sect_properties)

#####

dataset$durationgrp[dataset$DURATIONCHESTPAIN>=1 & dataset$DURATIONCHESTPAIN<3]<-1

dataset$durationgrp[dataset$DURATIONCHESTPAIN>=3 & dataset$DURATIONCHESTPAIN<=12]<-2

dataset$durationgrp[dataset$DURATIONCHESTPAIN>12 & dataset$DURATIONCHESTPAIN<=36]<-3

dataset$durationgrp=factor(dataset$durationgrp,levels = c(1:3),

labels = c("< 3 hours","3-12 hours","> 12 hours"))

names(dataset)[names(dataset)=="ORGAN SUPPORT"]<-"ORGAN"

names(dataset)[names(dataset)=="MECHANICAL VENTILATION"]<-"MECHANICAL"

dataset|>

dplyr::select(ORGAN,NIV,INOTROPES,HEMODIALYSIS,MECHANICAL,OUTCOME)|>

tbl_summary(by=OUTCOME,

missing='no',

digits = list(all_continuous() ~ 1,

all_categorical()~c(0,1)),

label = c(ORGAN~"ORGAN SUPPORT",

MECHANICAL~"MECHANICAL VENTILATION"))|>

modify_header(label ~ "**Variable**")|>

modify_caption("**Table 3: Other factors**")|>

bold_labels()|>

add_overall()|>

add_p(pvalue_fun = ~ style_pvalue(.x, digits = 2))|>

modify_header(label ~ "**Variable**") %>%

modify_spanning_header(c("stat_1", "stat_2") ~ "**Outcome**") %>%

modify_footnote(

all_stat_cols() ~ "Frequency (%)") %>%

as_flex_table()|>

flextable::save_as_docx(demo,path = ("C:\\Users\\James\\OneDrive\\Documents\\residents\\nadeem\\ICUdata\\Table 3.docx"), pr_section = sect_properties)

##########

dataset$LOS1=as.numeric(dataset$LOS)

dataset|>

dplyr::select(AGE,agegrp,BMI,ETHNICITY,GENDER,DIAGNOSIS1,DURATIONCHESTPAIN,

KILLIPCALSSIFICATIN,TROPS,TOTALCHOLESTROL,

HDL,LDL,TG,CREATININE,HB,PLT,CRP,ECG,DIAGNOSIS2,FIDNINGS,CULPRITVESSELPATTERN,

NONASCVD,CULPRIT,NONCULPRIT,LOS1,OUTCOME,LVEF,DSYFUNCTION,RWMA,LVTHROMBUS)|>

tbl_summary(by=DIAGNOSIS1,

label = c(LOS1~"Length of stay",

TROPS~"TROPONIN",

FIDNINGS~"FINDINGS",

NONASCVD~"NON-ASCVD",

NONCULPRIT~"NON-CULPRIT",

CULPRITVESSELPATTERN~"CULPRIT VESSEL PATTERN"),

missing='no',

digits = list(all_continuous() ~ 1,

all_categorical()~c(0,1)))|>

add_overall()|>

add_p(pvalue_fun = ~ style_pvalue(.x, digits = 2))|>

modify_footnote(

all_stat_cols() ~ "Median (IQR) or Frequency (%)")|>

modify_header(label ~ "**Variable**")|>

modify_spanning_header(c("stat_1", "stat_2") ~ "**Diagnosis**")|>

modify_caption("**Table 4: Comparative Characteristics**")|>

bold_labels()|>

as_flex_table()|>

flextable::save_as_docx(demo,path = ("C:\\Users\\James\\OneDrive\\Documents\\residents\\nadeem\\ICUdata\\Table 4.docx"), pr_section = sect_properties)

####

dataset|>

dplyr::select(INTEGRILLIN,NSTEMIAPPROACH,INTERVENTION,DIAGNOSIS1)|>

filter(DIAGNOSIS1=="NSTE-ACS")|>

tbl_summary(by=DIAGNOSIS1,

label = c(NSTEMIAPPROACH~"Type of intervention",

INTERVENTION~"Intervention"),

digits = list(all_continuous() ~ 1,

all_categorical()~c(0,1)))|>

bold_labels()|>

as_flex_table()|>

flextable::save_as_docx(demo,path = ("C:\\Users\\James\\OneDrive\\Documents\\residents\\nadeem\\ICUdata\\Table 5.docx"), pr_section = sect_properties)

####

dataset|>

dplyr::select(INTEGRILLIN,INTERVENTION,DIAGNOSIS1)|>

filter(DIAGNOSIS1=="STEMI")|>

tbl_summary(by=DIAGNOSIS1,

label = c(INTERVENTION~"Intervention"),

digits = list(all_continuous() ~ 1,

all_categorical()~c(0,1)))|>

bold_labels()|>

as_flex_table()|>

flextable::save_as_docx(demo,path = ("C:\\Users\\James\\OneDrive\\Documents\\residents\\nadeem\\ICUdata\\Table 6.docx"), pr_section = sect_properties)

####getting the means

dataset|>

dplyr::select(TROPS,TOTALCHOLESTROL,

HDL,LDL,TG,CREATININE,HB,PLT,CRP,LVEF,DURATIONCHESTPAIN,HBA1C,DIAGNOSIS1)|>

tbl_summary(by=DIAGNOSIS1,

missing = 'no',

statistic = list(

all_continuous() ~ "{mean} ({sd})",

all_categorical() ~ "{n} / {N} ({p}%)"),

digits = list(all_continuous() ~ 1,

all_categorical()~c(0,1)))|>

add_p(test = everything() ~ "t.test",

pvalue_fun = ~ style_pvalue(.x, digits = 2),

test.args = all_tests("t.test") ~ list(var.equal = FALSE))|>

add_overall()|>

as_flex_table()|>

flextable::save_as_docx(demo,path = ("C:\\Users\\James\\OneDrive\\Documents\\residents\\nadeem\\ICUdata\\Table 7.docx"), pr_section = sect_properties)

####additional analysis

barplot(FIDNINGS~GENDER)

print(ctable(dataset$FIDNINGS,dataset$GENDER,useNA = 'no'))

boxplot(dataset$TROPS~dataset$DIAGNOSIS1,xlab = "Diagnosis",ylab = "Troponin values")

######Table 5

names(dataset)[names(dataset)=="SYSTOLIC BP"]<-"SBP"

names(dataset)[names(dataset)=="DIASTOLIC BP"]<-"DBP"

dataset|>

dplyr::select(SBP,DBP,PR,RR,TEMP,OUTCOME)|>

tbl_summary(by=OUTCOME,

missing = 'no',

statistic = list(

all_continuous() ~ "{mean} ({sd})",

all_categorical() ~ "{n} / {N} ({p}%)"),

digits = list(all_continuous() ~ 1,

all_categorical()~c(0,1)))|>

add_p(test = everything() ~ "t.test",

pvalue_fun = ~ style_pvalue(.x, digits = 2),

test.args = all_tests("t.test") ~ list(var.equal = FALSE))|>

add_overall()|>

as_flex_table()|>

flextable::save_as_docx(demo,path = ("C:\\Users\\James\\OneDrive\\Documents\\residents\\nadeem\\ICUdata\\Table 5.docx"), pr_section = sect_properties)

######################plots

par(mfrow=c(1,3))

boxplot(SBP~OUTCOME,data=dataset)

boxplot(DBP~OUTCOME,data=dataset)

boxplot(PR~OUTCOME,data=dataset)

par(mfrow=c(1,2))

boxplot(RR~OUTCOME,data=dataset)

boxplot(TEMP~OUTCOME,data=dataset)

dev.off()

boxplot(CRP~OUTCOME,data=dataset)

####scatterplot

# Calculate the correlation coefficient and p-value

ggplot(dataset, aes(x = SBP, y = DBP, shape = OUTCOME, color = OUTCOME)) +

geom_point() +

geom_smooth(method = lm, se = FALSE, fullrange = TRUE) +

xlim(0,270)+

ylim(0,180)+

annotate("text", x=200, y=30, label= "r=0.81; p<0.001")+

theme_classic()

####Table 6

dataset$CREATININE=as.numeric(dataset$CREATININE)

dataset$PROCALCITONIN=as.numeric(dataset$PROCALCITONIN)

dataset|>

dplyr::select(CRP,PROCALCITONIN,CREATININE,OUTCOME)|>

tbl_summary(by=OUTCOME,

missing = 'no',

statistic = list(

all_continuous() ~ "{mean} ({sd})",

all_categorical() ~ "{n} / {N} ({p}%)"),

digits = list(all_continuous() ~ 1,

all_categorical()~c(0,1)))|>

add_p(test = everything() ~ "t.test",

pvalue_fun = ~ style_pvalue(.x, digits = 2),

test.args = all_tests("t.test") ~ list(var.equal = FALSE))|>

add_overall()|>

as_flex_table()|>

flextable::save_as_docx(demo,path = ("C:\\Users\\James\\OneDrive\\Documents\\residents\\nadeem\\ICUdata\\Table 6.docx"), pr_section = sect_properties)

##################

names(dataset)[names(dataset)=="LOS ICU (D)"]<-"LOS"

names(dataset)[names(dataset)=="DIAGNOSIS - CATEGORY"]<-"DIAGNOSISCATEGORY"

boxplot(dataset$LOS~dataset$OUTCOME,

ylab = "Length of hospital stay (days)",

xlab = "Survival status")

dataset|>

dplyr::select(DIAGNOSISCATEGORY,OUTCOME)|>

tbl_summary(by=OUTCOME,

missing = 'no',

digits = list(all_continuous() ~ 1,

all_categorical()~c(0,1)))|>

add_overall()|>

modify_header(label ~ "**DIAGNOSIS CATEGORY**")|>

as_flex_table()|>

flextable::save_as_docx(demo,path = ("C:\\Users\\James\\OneDrive\\Documents\\residents\\nadeem\\ICUdata\\Table 7.docx"), pr_section = sect_properties)

######################################

####test of proportions

prop.test(x = c(18, 92), n = c(128, 589))

prop.test(x = c(3, 46), n = c(128, 589))

prop.test(x = c(13, 46), n = c(128, 589))

prop.test(x = c(4, 9), n = c(128, 589))

prop.test(x = c(40, 135), n = c(128, 589))

prop.test(x = c(12, 59), n = c(128, 589))

prop.test(x = c(12, 99), n = c(128, 589))

prop.test(x = c(13, 16), n = c(128, 589))

prop.test(x = c(0, 6), n = c(128, 589))

prop.test(x = c(13, 65), n = c(128, 589))

prop.test(x = c(0, 12), n = c(128, 589))
